# *Cassida piperata* Hope, 1843 (Coleoptera, Chrysomelidae), a non-native beetle, new to Italy and Europe

**DOI:** 10.3897/BDJ.13.e177770

**Published:** 2025-12-22

**Authors:** Enrico Ruzzier, Marco Uliana

**Affiliations:** 1 Roma Tre University, Department of Science, Rome, Italy Roma Tre University, Department of Science Rome Italy; 2 National Biodiversity Future Center (NBFC), Palermo, Italy National Biodiversity Future Center (NBFC) Palermo Italy; 3 World Biodiversity Association Onlus, Verona, Italy World Biodiversity Association Onlus Verona Italy; 4 Museo di Storia Naturale di Venezia Giancarlo Ligabue, Venezia, Italy Museo di Storia Naturale di Venezia Giancarlo Ligabue Venezia Italy

**Keywords:** faunistic, introduction, leaf beetle, monitoring, non-native species

## Abstract

**Background:**

Maintaining sustained vigilance over the introduction and spread of non-native species is of critical importance.

**New information:**

*Cassida piperata*, a leaf beetle native to the East Palaearctic and Indomalayan Regions, has been repeatedly recorded in northern Italy since 2021, where it appears to be established as a non-native species new to Europe. Its local occurrence is discussed, based on records obtained by the authors and from citizen-science web platforms and its potential for further spread in Europe is evaluated through bioclimatic modelling.

## Introduction

In an increasingly globalised world, sustained vigilance towards the introduction and spread of non-native species is crucial (Banks et al. 2015, Seebens et al. 2018). Effective management of biological invasions relies not only on early detection, but also on the continuous production of faunistic records and the regular updating of reliable, accessible distributional data (Groom et al. 2015, Wallace et al. 2020). These activities are fundamental in supporting prevention strategies and in enhancing understanding of the dynamics of species introductions. Amongst European countries, Italy stands out for the high number of intercepted, adventive and established non-native Coleoptera species (e.g. Cocquempot and Lindelöw 2010, Denux and Zagatti 2010, Kirkendall and Faccoli 2010, Roy and Migeon 2010, Sauvard et al. 2010, Jelínek et al. 2016, Montagna et al. 2016, Binazzi et al. 2019, Forbicioni 2019, Ruzzier and Colla 2019, Ruzzier et al. 2020a, Ruzzier et al. 2020b, Ruzzier et al. 2021, Marchioro et al. 2022, Ruzzier et al. 2022, Mola et al. 2023, Ruzzier et al. 2023a, Ruzzier et al. 2023b, Ruzzier et al. 2023c). This trend can be largely attributed to Italy’s central position within the Mediterranean Basin and its strategic role in international trade, which makes it a key transit hub and potential gateway for non-native species entering the European continent (Roques 2010, Rassati et al. 2014). Chrysomelidae (Coleoptera, Chrysomeloidea) is one of the largest and most diverse families of phytophagous beetles, some of which pose significant ecological and economic threats, particularly as pests of cultivated plants in invaded areas (Jolivet et al. 2005). In Europe and, specifically, in Italy, the group includes several non-native species that have been documented over the years, with some successfully acclimatising (Beenen 2005, Beenen and Roques 2010, Clark et al. 2014, Yus-Ramos et al. 2014, Montagna et al. 2016), as well as native species for which a significant range expansion has been recently observed (Uliana and Busato 2022). The present contribution provides the first European records of *Cassida piperata* Hope, 1843 (Coleoptera, Chrysomelidae, Cassidinae), a non-native leaf beetle of East Palaearctic and Indomalayan origin and assesses its potential for further spread through bioclimatic modelling. Available records, deriving both from our field observations and citizen-science records from Italy suggest that the species is successfully established in the region.

## Materials and methods

**Figure 1.**
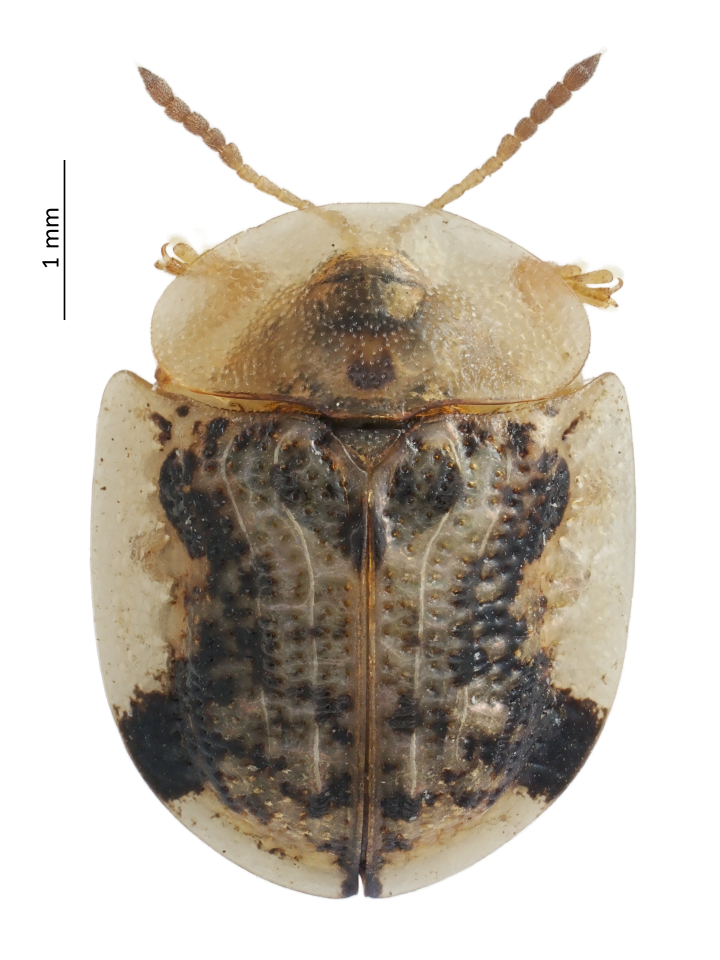
*Cassida piperata*, habitus of a specimen collected in Codevigo, NE Italy.

Specimens from Codevigo were directly examined and identified according to Warchałowski (2010); the identification was also confirmed by specialist Lukáš Sekerka (National Museum, Prague, Czechia), based on high-resolution photos. All web-based records, some of which had been previously identified by specialists, were re-evaluated for confirmation based on the species’ habitus (Fig. 1), whose colouration pattern is diagnostic in distinguishing it from other European *Cassida*. In addition, the photographs were also compared with the habitus of *C. japana* Baly, 1874 (www.cassidae.uni.wroc.pl/katalog%20internetowy/cassidajapana.htm), another Oriental species with which *C. piperata* can occasionally be confused. The list of records herein provided includes only those originating from the authors or provided by their colleagues, as well as records obtained from web forums not linked to GBIF (www.naturamediterraneo.com; www.entomologiitaliani.net). Italian records from iNaturalist (18 research-grade observations, accessed on 8 November 2025) were used for distribution map realisation and are also listed here (Suppl. material 1).

### Data preparation and bioclimatic suitability modelling

Global occurrences of *C. piperata* were downloaded from the Global Biodiversity Information Facility using the *rgibf* R package (Chamberlain et al. 2024), from iNaturalist via the *spocc* R package (Owens et al. 2024). The data were checked for all potential issues (e.g. duplicate records, misidentifications etc.). Before modelling, the occurrences recurring in the calibration area were thinned through the function “ensemble.spatialThin” of the package *BiodiversityR* (Kindt and Coe 2005), with a thinning parameter of 5.0 km in R to exclude duplicated data and remove occurrences within the same raster cell. Pseudoabsences (PAs) were distributed across a geographical extent (80°E–115°E; 15°N–72°N) encompassing the native range of *C. piperata*. Within this extent, PAs were allocated with a density inversely proportional to the density of *C. piperata* occurrences (see Ruzzier et al. (2025a), Ruzzier et al. (2025b)). To estimate occurrence density, presence records were converted into a georeferenced point pattern using the as.ppp function and interpolated via kernel density estimation (density.ppp), with the kernel bandwidth determined by bw.ppl. All functions are part of the *spatstat* R package (Baddeley and Turner 2005). The natural logarithm of the resulting kernel density surface, applied to mitigate the influence of oversampling-related density peaks, was used as a proxy for species abundance across *C. piperata's* native range and adjacent regions. The model calibration area for bioclimatic suitability was defined as regions where kernel density exceeded 0.1 points per 100 km². Bioclimatic layers averaged for the years 1970–2000 were downloaded from WorldClim version 2.1 at a spatial resolution of 0.00833°. These layers were then aggregated using the *terra* R package (Hijmans 2024) to a resolution of 0.04166667° (approximately 5 km at the Equator). From all available layers, a subset was subsequently selected and tested for multicollinearity (Pearson’s correlation coefficient |r| > 0.7) across the native range of *C. piperata* (range extent: 80°E–115°E; 15°N–72°N). The retained bioclimatic layers (BIO5 – maximum temperature of the warmest month, BIO6 – minimum temperature of the coldest month, BIO13 – precipitation of the wettest month and BIO14 – precipitation of the driest month) were then used in the modelling phase; in addition, layers BIO13 and BIO14 were log-transformed. The modelling procedure was conducted using the *biomod2* package (version 4.2-5-2; Thuiller et al. (2024)). The following algorithms were employed: Artificial Neural Network (ANN), Generalised Additive Model (GAM), Generalised Boosting Model (GBM), Random Forest (RF) and Maximum Entropy (MAXNET), all implemented under the “bigboss” settings (Thuiller et al. 2024). A total of five sets of pseudo-absences were generated for the species distribution modelling (SDM). Due to the lack of external data for independent validation, model accuracy was assessed via a 5-fold cross-validation procedure, using 80% of the data for model training and the remaining 20% for validation (Ruzzier et al. 2024). Each model, derived from the bioclimatic suitability modelling, was evaluated using the area under the receiver operating characteristic curve (AUC) and the True Skill Statistic (TSS) (Threshold: 0.8 for ROC; 0.6 for TSS) (Suppl. material 2).

## Taxon treatments

### Cassida piperata

Hope, 1842

8CA7D5C9-631B-52F3-B058-0CAC6B3EAC27

#### Materials

- **Type status:** Other material. **Occurrence:** occurrenceRemarks: external wall of a building; recordedBy: Marco Uliana; individualCount: 1; lifeStage: adult; establishmentMeans: introduced; occurrenceStatus: present; occurrenceID: 95FAAAE8-D451-5C8D-84C9-4132743D5831; **Taxon:** taxonID: https://www.gbif.org/species/5876242; scientificName: Cassida piperata Hope, 1843; acceptedNameUsage: Cassida piperata Hope, 1842; parentNameUsage: Chrysomelidae; kingdom: Animalia; phylum: Arthropoda; class: Insecta; order: Coleoptera; family: Chrysomelidae; genus: Cassida; specificEpithet: piperata; taxonRank: species; scientificNameAuthorship: Hope, 1843; **Location:** country: Italy; stateProvince: Veneto; county: Padova; municipality: Codevigo; locality: Rosara; verbatimElevation: 0 m; decimalLatitude: 45.2928; decimalLongitude: 12.1001; coordinateUncertaintyInMeters: 5; georeferencedBy: Marco Uliana; georeferenceSources: GoogleMaps; **Identification:** identifiedBy: Marco Uliana; dateIdentified: 2025; **Event:** samplingProtocol: visual search; year: 2024; month: 9; day: 12; **Record Level:** institutionCode: MSNVE; collectionCode: Insects; basisOfRecord: PreservedSpecimen
- **Type status:** Other material. **Occurrence:** occurrenceRemarks: overwintering on a log pile; recordedBy: Marco Uliana; individualCount: 6; lifeStage: adult; establishmentMeans: introduced; occurrenceStatus: present; occurrenceID: BACB9431-807B-5F03-96B6-A384C9A07348; **Taxon:** taxonID: https://www.gbif.org/species/5876242; scientificName: Cassida piperata Hope, 1843; acceptedNameUsage: Cassida piperata Hope, 1842; parentNameUsage: Chrysomelidae; kingdom: Animalia; phylum: Arthropoda; class: Insecta; order: Coleoptera; family: Chrysomelidae; genus: Cassida; specificEpithet: piperata; taxonRank: species; scientificNameAuthorship: Hope, 1843; **Location:** country: Italy; stateProvince: Veneto; county: Padova; municipality: Codevigo; locality: Rosara; verbatimElevation: 0 m; decimalLatitude: 45.2927; decimalLongitude: 12.1005; coordinateUncertaintyInMeters: 5; georeferencedBy: Marco Uliana; georeferenceSources: GoogleMaps; **Identification:** identifiedBy: Marco Uliana; dateIdentified: 2025; **Event:** samplingProtocol: visual search; year: 2024; month: 11; day: 7; **Record Level:** institutionCode: MSNVE; collectionCode: Insects; basisOfRecord: PreservedSpecimen
- **Type status:** Other material. **Occurrence:** occurrenceRemarks: external wall of a building; recordedBy: Marco Uliana; individualCount: 1; lifeStage: adult; establishmentMeans: introduced; occurrenceStatus: present; occurrenceID: A2897F0D-B6B5-5630-8D42-B7B1CC8DC164; **Taxon:** taxonID: https://www.gbif.org/species/5876242; scientificName: Cassida piperata Hope, 1843; acceptedNameUsage: Cassida piperata Hope, 1842; parentNameUsage: Chrysomelidae; kingdom: Animalia; phylum: Arthropoda; class: Insecta; order: Coleoptera; family: Chrysomelidae; genus: Cassida; specificEpithet: piperata; taxonRank: species; scientificNameAuthorship: Hope, 1843; **Location:** country: Italy; stateProvince: Veneto; county: Padova; municipality: Codevigo; locality: Rosara; verbatimElevation: 0 m; decimalLatitude: 45.2928; decimalLongitude: 12.1001; coordinateUncertaintyInMeters: 5; georeferencedBy: Marco Uliana; georeferenceSources: GoogleMaps; **Identification:** identifiedBy: Marco Uliana; dateIdentified: 2025; **Event:** samplingProtocol: visual search; year: 2025; month: 10; day: 13; **Record Level:** institutionCode: MSNVE; collectionCode: Insects; basisOfRecord: PreservedSpecimen
- **Type status:** Other material. **Occurrence:** occurrenceRemarks: in litter; recordedBy: Marco Uliana; individualCount: 1; lifeStage: adult; establishmentMeans: introduced; occurrenceStatus: present; occurrenceID: E7C72424-12E8-5FC2-8A00-B6CB56E408B7; **Taxon:** taxonID: https://www.gbif.org/species/5876242; scientificName: Cassida piperata Hope, 1843; acceptedNameUsage: Cassida piperata Hope, 1842; parentNameUsage: Chrysomelidae; kingdom: Animalia; phylum: Arthropoda; class: Insecta; order: Coleoptera; family: Chrysomelidae; genus: Cassida; specificEpithet: piperata; taxonRank: species; scientificNameAuthorship: Hope, 1843; **Location:** country: Italy; stateProvince: Veneto; county: Padova; municipality: Codevigo; locality: Rosara; verbatimElevation: 0 m; decimalLatitude: 45.2927; decimalLongitude: 12.1001; coordinateUncertaintyInMeters: 5; georeferencedBy: Marco Uliana; georeferenceSources: GoogleMaps; **Identification:** identifiedBy: Marco Uliana; dateIdentified: 2025; **Event:** samplingProtocol: visual search; year: 2025; month: 11; day: 2; **Record Level:** institutionCode: MSNVE; collectionCode: Insects; basisOfRecord: PreservedSpecimen
- **Type status:** Other material. **Occurrence:** recordedBy: Davide Perdersoli; individualCount: 1; lifeStage: adult; establishmentMeans: introduced; occurrenceStatus: present; occurrenceID: 6D75D987-0B1E-59F3-8D9D-02A36C0617AC; **Taxon:** taxonID: https://www.gbif.org/species/5876242; scientificName: Cassida piperata Hope, 1843; acceptedNameUsage: Cassida piperata Hope, 1842; parentNameUsage: Chrysomelidae; kingdom: Animalia; phylum: Arthropoda; class: Insecta; order: Coleoptera; family: Chrysomelidae; genus: Cassida; specificEpithet: piperata; taxonRank: species; scientificNameAuthorship: Hope, 1843; **Location:** country: Italy; stateProvince: Lombardia; county: Bergamo; municipality: Rogno; locality: bank of Oglio river, Colombine; verbatimElevation: 200 m; verbatimCoordinates: 45.8515, 10.1378; decimalLatitude: 45.8515; decimalLongitude: 10.1378; coordinateUncertaintyInMeters: 10; georeferencedBy: Marco Uliana; georeferenceSources: GoogleMaps; **Identification:** identifiedBy: Davide Pedersoli, Marco Uliana; dateIdentified: 2025; **Event:** year: 2025; month: 5; day: 13; **Record Level:** collectionID: coll. Davide Pedersoli; basisOfRecord: PreservedSpecimen
- **Type status:** Other material. **Occurrence:** recordedBy: Davide Perdersoli; individualCount: 6; lifeStage: adult; establishmentMeans: introduced; occurrenceStatus: present; occurrenceID: 38499912-9D0A-5D95-B941-9E5A96E5134A; **Taxon:** taxonID: https://www.gbif.org/species/5876242; scientificName: Cassida piperata Hope, 1843; acceptedNameUsage: Cassida piperata Hope, 1842; parentNameUsage: Chrysomelidae; kingdom: Animalia; phylum: Arthropoda; class: Insecta; order: Coleoptera; family: Chrysomelidae; genus: Cassida; specificEpithet: piperata; taxonRank: species; scientificNameAuthorship: Hope, 1843; **Location:** country: Italy; stateProvince: Lombardia; county: Bergamo; municipality: Rogno; locality: bank of Oglio river; verbatimElevation: 195 m; verbatimCoordinates: 45.8422, 10.1262; decimalLatitude: 45.8422; decimalLongitude: 10.1262; coordinateUncertaintyInMeters: 10; georeferencedBy: Marco Uliana; georeferenceSources: GoogleMaps; **Identification:** identifiedBy: Davide Pedersoli, Marco Uliana; dateIdentified: 2025; **Event:** year: 2025; month: 8; day: 20; **Record Level:** collectionID: coll. Davide Pedersoli; basisOfRecord: PreservedSpecimen
- **Type status:** Other material. **Occurrence:** recordedBy: Riccardo Panzeri; individualCount: 1; lifeStage: adult; establishmentMeans: introduced; occurrenceStatus: present; associatedReferences: http://www.entomologiitaliani.net/public/forum/phpBB3/viewtopic.php?f=146&t=98742; occurrenceID: 310DD9F1-2DFE-5E5B-85AE-77C8EAE59065; **Taxon:** taxonID: https://www.gbif.org/species/5876242; scientificName: Cassida piperata Hope, 1843; acceptedNameUsage: Cassida piperata Hope, 1842; parentNameUsage: Chrysomelidae; kingdom: Animalia; phylum: Arthropoda; class: Insecta; order: Coleoptera; family: Chrysomelidae; genus: Cassida; specificEpithet: piperata; taxonRank: species; scientificNameAuthorship: Hope, 1843; **Location:** country: Italy; stateProvince: Lombardia; county: Lecco; municipality: Verderio; locality: Verderio; decimalLatitude: 45.66; decimalLongitude: 9.44; coordinateUncertaintyInMeters: 1000; georeferencedBy: Marco Uliana; georeferenceSources: GoogleMaps; **Identification:** identifiedBy: Lech Borowiec, Marco Uliana; dateIdentified: 2025; **Event:** year: 2022; month: 5; **Record Level:** collectionID: coll. Riccardo Panzeri; basisOfRecord: PreservedSpecimen
- **Type status:** Other material. **Occurrence:** occurrenceRemarks: anthropized area, on Amaranthus retroflexus; recordedBy: Boris Ziliotto; individualCount: 4; lifeStage: adult; behavior: mating; establishmentMeans: introduced; occurrenceStatus: present; associatedReferences: https://www.naturamediterraneo.com/forum/topic.asp?TOPIC_ID=334231; occurrenceID: 8C37D3AE-AB0D-5A72-93F2-882A1A695B6A; **Taxon:** taxonID: https://www.gbif.org/species/5876242; scientificName: Cassida piperata Hope, 1843; acceptedNameUsage: Cassida piperata Hope, 1842; parentNameUsage: Chrysomelidae; kingdom: Animalia; phylum: Arthropoda; class: Insecta; order: Coleoptera; family: Chrysomelidae; genus: Cassida; specificEpithet: piperata; taxonRank: species; scientificNameAuthorship: Hope, 1843; **Location:** country: Italy; stateProvince: Veneto; county: Padova; municipality: Vigonza; decimalLatitude: 45.45; decimalLongitude: 11.98; coordinateUncertaintyInMeters: 2000; georeferencedBy: Marco Uliana; georeferenceSources: GoogleMaps; **Identification:** identifiedBy: Lech Borowiec, Marco Uliana; dateIdentified: 2025; **Event:** eventDate: 2021-07-17/31; year: 2021; month: 7; day: 17; **Record Level:** basisOfRecord: HumanObservation
- **Type status:** Other material. **Occurrence:** occurrenceRemarks: anthropized area, on Amaranthus retroflexus; recordedBy: Boris Ziliotto; individualCount: 1; lifeStage: larva; establishmentMeans: introduced; occurrenceStatus: present; associatedReferences: https://www.naturamediterraneo.com/forum/topic.asp?TOPIC_ID=334231; occurrenceID: 528F1F20-F520-50EC-BA2A-0FB4CD6D0789; **Taxon:** taxonID: https://www.gbif.org/species/5876242; scientificName: Cassida piperata Hope, 1843; acceptedNameUsage: Cassida piperata Hope, 1842; parentNameUsage: Chrysomelidae; kingdom: Animalia; phylum: Arthropoda; class: Insecta; order: Coleoptera; family: Chrysomelidae; genus: Cassida; specificEpithet: piperata; taxonRank: species; scientificNameAuthorship: Hope, 1843; **Location:** country: Italy; stateProvince: Veneto; county: Padova; municipality: Vigonza; decimalLatitude: 45.45; decimalLongitude: 11.98; coordinateUncertaintyInMeters: 2000; georeferencedBy: Marco Uliana; georeferenceSources: GoogleMaps; **Identification:** identifiedBy: Lech Borowiec, Marco Uliana; dateIdentified: 2025; **Event:** year: 2021; month: 7; day: 31; **Record Level:** basisOfRecord: HumanObservation

#### Taxon discussion

**Figure 2.**
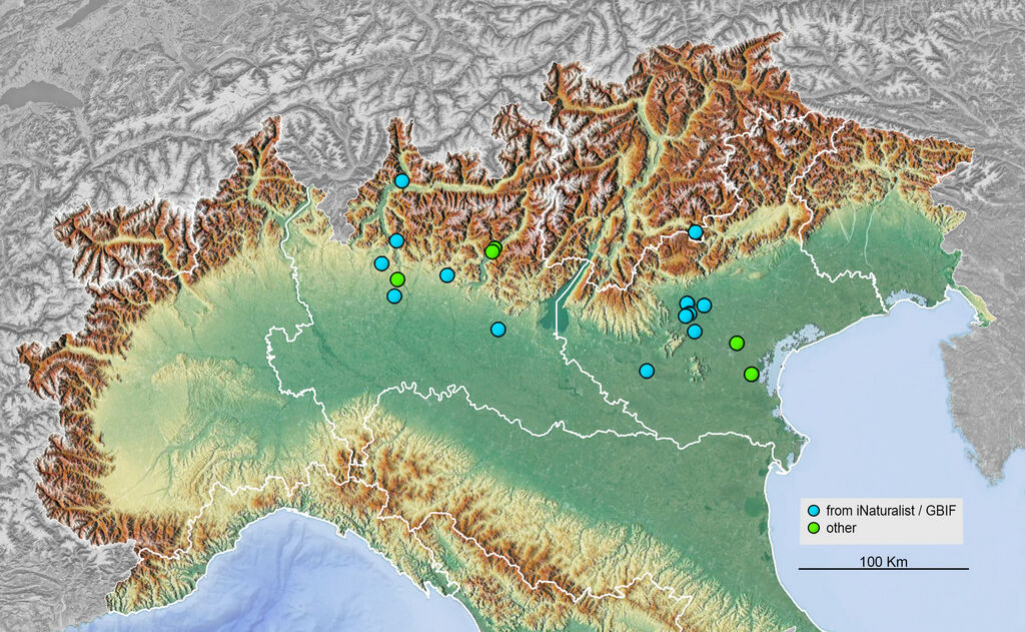
Occurrences of *Cassida piperata* in Italy.

*Cassida piperata* Hope, 1842 (Coleoptera, Chrysomelidae, Cassidinae) is a species native to the Eastern Palaearctic and Indomalayan Regions (Borowiec 1990, Bezděk and Sekerka 2024). It is widely distributed across Southeast Asia, having been recorded from China (Anhui, Beijing, Chongqing, Fujian, Gansu, Guangdong, Guizhou, Guangxi, Hainan, Hebei, Heilongjiang, Henan, Hubei, Hunan, Jiangsu, Jiangxi, Jilin, Liaoning, Ningxia, Qinghai, Shaanxi, Shandong, Shanxi, Sichuan, Taiwan, Tianjin, Xinjiang, Xizang (Tibet), Yunnan, Zhejiang, Hong Kong, Macao), North and South Korea, Japan, the Philippines, Vietnam and the Russian Far East (Qi et al. 2008, Sekerka and Świetojańska 2024). Despite this broad distribution, the species is generally regarded as rare throughout most of its range (Zhang et al. 2008). The species is currently established in North America (iNaturalist) and it is here newly recorded in Italy and Europe. Italian records are mapped in Fig. 2.

## Discussion

**Figure 3.**
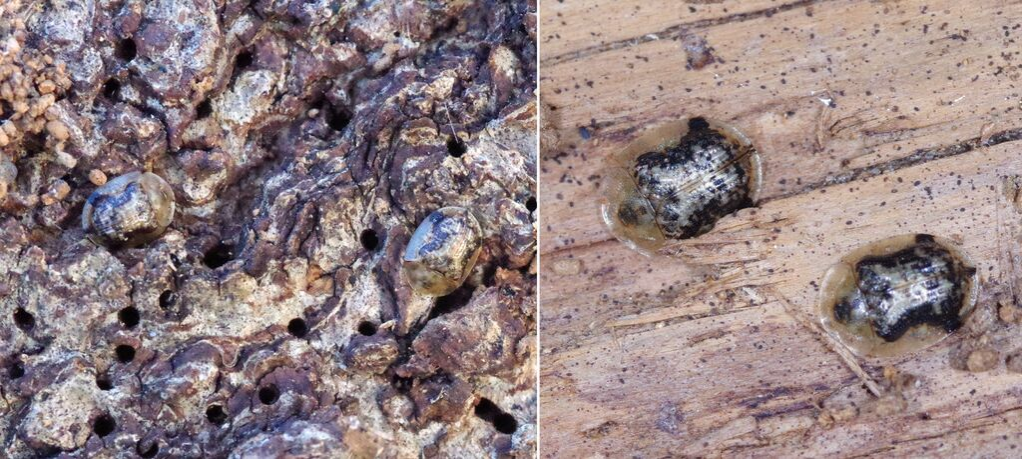
Adults of *Cassida piperata* overwintering on logs observed at Codevigo, Italy, December 2024.

*Cassida piperata* is a stenophagous tortoise beetle that feeds on the leaves of several species within the families Amaranthaceae and Chenopodiaceae (Dai et al. 2013, Nagasawa and Matsuda 2015). Documented host plants include *Alternanthera sessilis*, *Achyranthes* spp., *Amaranthus* spp., *Celosia* spp., *Chenopodium* spp., *Beta* spp. and *Atriplex* spp. (Kimoto 1966). Due to its relative trophic adaptability, *C. piperata* has been evaluated as a potential biological control agent against multiple invasive plant species in China. Research has provided valuable insights into both the potential and limitations of *C. piperata* (Coleoptera, Chrysomelidae, Cassidinae) for controlling *Alternanthera philoxeroides* (alligator weed), indicating, however, that its control efficacy remains limited (Lu and Ding 2011, He et al. 2021, Liu et al. 2021). The ability of *C. piperata* to exploit both native and invasive host plants as trophic and reproductive resources highlights a high degree of ecological plasticity, allowing it to adapt and thrive in newly-introduced environments; this plasticity is further confirmed by the occurrence of established populations of this species in both North America and Europe. *Cassida piperata* has been present in North America since at least 2007 (Nanz 2007) and is now widely established throughout the eastern and central United States, as well as in the southernmost parts of Canada (see iNaturalist). Despite the widespread distribution of the species and its relatively long-established introduction, the North American entomological community has so far paid little attention to it (BugGuide). More recently, the same species has also been recorded in Europe, specifically in Italy, where several records have emerged from direct observations and citizen-science platforms, such as iNaturalist. The first observation of this species in Italy (and therefore in Europe) dates back to 2020 in Grigno (Trentino-Alto Adige, northern Italy) (iNaturalist record). It was subsequently recorded in Vigonza (Veneto, northern Italy) in 2021, with multiple specimens observed (naturamediterraneo.com) and later documented in several provinces of Lombardy, Trentino-Alto Adige and Veneto between 2023 and 2025. The frequency and spatial distribution of observations indicate that the species has successfully established itself in northern Italy, at least within the Po Valley. Moreover, the occurrence of reproductively active populations is further supported by photographic evidence of larvae and mating adults on *Amaranthus retroflexus* in Vigonza (Veneto), as well as by repeated occurrences of the species in a context where passive translocation is implausible (Rosara, Veneto). All adults recorded in the latest locality were found in a house garden surrounded by farmland within an extensive agricultural landscape. Just a few tens of metres from the observation sites, crops such as soybean, corn and wheat were regularly associated with dense growths of *Amaranthus retroflexus* and *Chenopodium album*, one of the main host plants of the species in its native range. Given the otherwise limited occurrence of potential host plants in nearby semi-natural areas, we hypothesised that at least one of *C. album* or *A. retroflexus* could serve as a host for *C. piperata* in that agricultural landscape. Repeated searches conducted between June and October aimed to test this hypothesis. Net sweeping and visual inspection of dense stands of these potential host plants at the margins of soybean fields, although yielding abundant phytophagous insects, produced no records of *C. piperata*. This absence may be related to the high density of the potential host plants, where a small number of *C. piperata* individuals could remain too scattered to be easily detected. It remains intriguing how *C. piperata* was first introduced from its native range into Europe and North America and how it subsequently spread so rapidly throughout the invaded areas. It is quite plausible that the species has been introduced and dispersed passively by humans, in particular through the transport of packaging materials and firewood. *Cassida piperata* is indeed able to overwinter as an adult (Wei et al. 2016) and it is not uncommon, as we have also observed, to find individuals overwintering beneath tree bark or amongst stacked firewood, where they may exhibit gregarious behaviour (Fig. 3). Adults overwintering in Codevigo, retained throughout the winter the ability to move and change position, relocating after disturbance.

**Figure 4.**
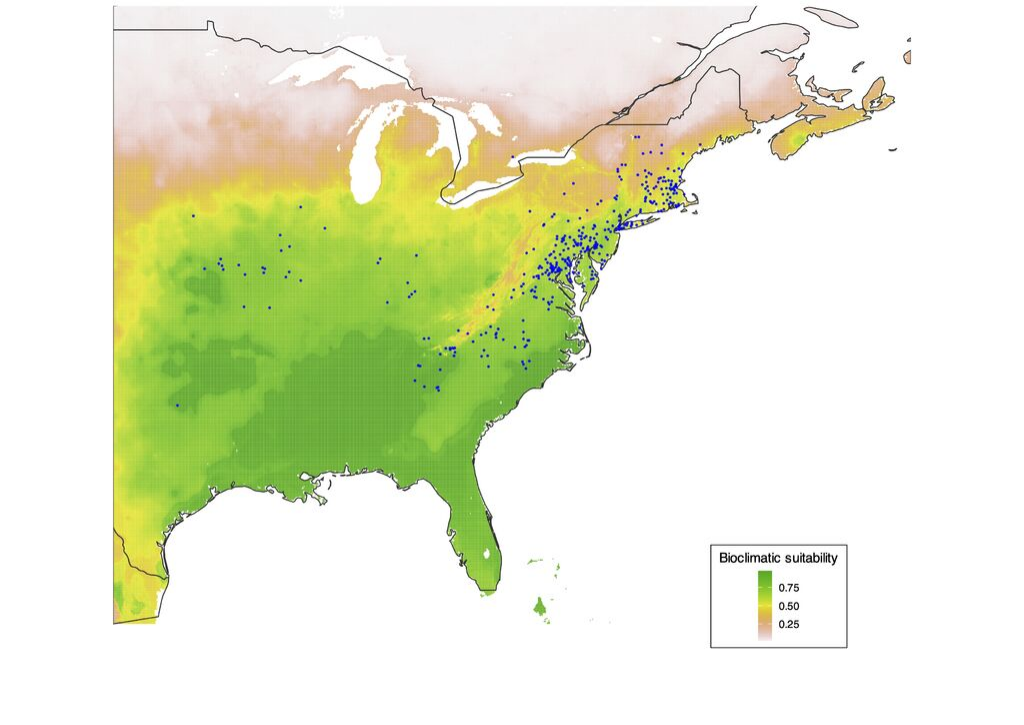
Suitability map of *Cassida piperata* in north-eastern North America. Blue points indicate *C. piperata* occurrences.

Currently, no data are available on the impact of this species on invaded ecosystems. However, preliminary observations suggest that *C. piperata*, at least in Europe, may preferentially utilise invasive plant species present in the region, such as *A. retroflexus*, as host plants. The occurrence of these plants may, in fact, have facilitated the successful establishment of this species and it is plausible that, in ecologically suitable areas, *C. piperata* could act as a potential natural control agent for certain invasive plant species. A more detailed investigation is, however, warranted, as Nagasawa and Matsuda suggest that chard, table beet and spinach may also serve as potential host plants for *C. piperata* (Nagasawa and Matsuda 2015). An intriguing aspect of the establishment of *Cassida piperata* in Europe lies in its interaction with its principal natural enemy, the parasitoid *Holcotetrastichus rhosaces* (Walker, 1839) (Hymenoptera, Eulophidae). This wasp, widely distributed in Europe, is known to parasitise several species of the genus *Cassida*. In Japan, *H. rhosaces* has been recorded from *C. nebulosa*, *C. piperata* and *C. japana* and field studies have shown that populations of both *C. nebulosa* and *C. piperata* were strongly regulated by this parasitoid (Nagasawa 2014). Species distribution models, based on the bioclimatic niche of *C. piperata* in its native range, indicate that its distribution is primarily influenced by the minimum temperature of the coldest month (BIO6; variable contribution: 0.223), the precipitation of the wettest month (BIO13; variable contribution: 0.219) and the maximum temperature of the warmest month (BIO5; variable contribution: 0.153). Specifically, suitable areas are characterised by winter temperatures not lower than -10°C, hot summers with temperatures exceeding 30°C and a wettest month precipitation of approximately 1,100 mm. These conditions correspond to a temperate-humid climate with warm summers and moderately cold winters, typical of continental or sub-continental temperate regions with sufficient water availability during the wet season. Projections of the distribution model on the invaded areas indicate two different scenarios: in North America (Fig. 4), most of the central and eastern United States, part of north-eastern Mexico and a small area of Canada near the Great Lakes appear to be highly suitable for *C. piperata*. The species is, in fact, present and successfully established in many regions of the eastern United States, where a natural expansion from the initial introduction sites is plausible. The records from the central States clearly indicate human-mediated translocations, which have likely resulted in the establishment of additional viable populations.

**Figure 5.**
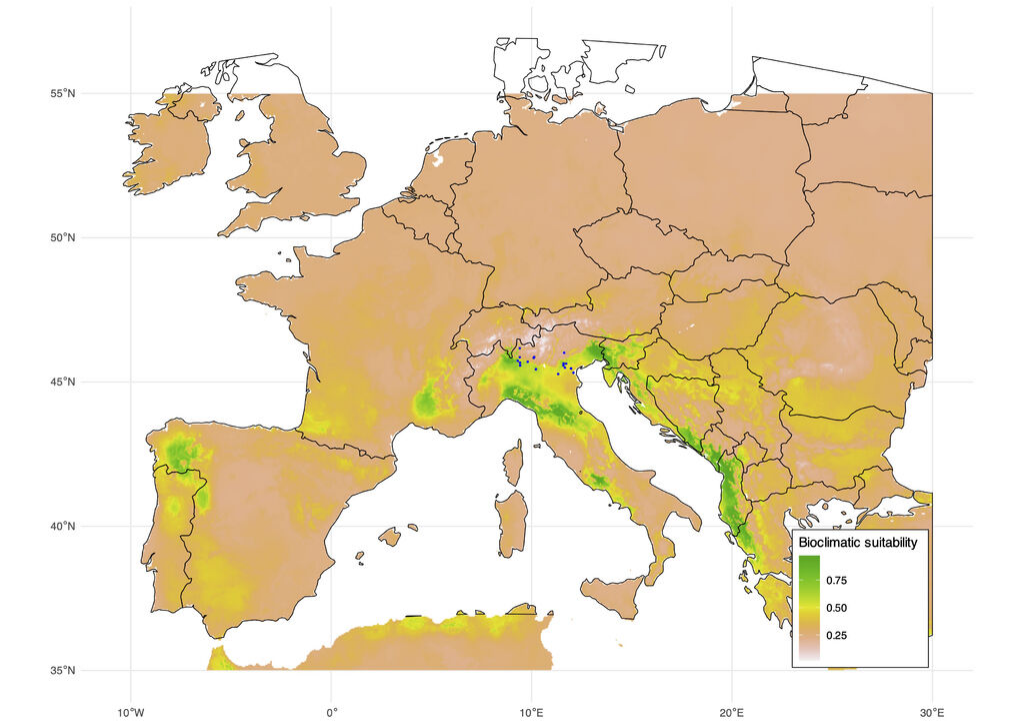
Suitability map of *Cassida piperata* in Europe. Blue points indicate *C. piperata* occurrences.

Unlike North America, most of Europe appears to be poorly suitable for the species, with only a few localised areas showing favourable conditions. These include northern Italy, particularly the Alpine foothills and the Apennine Region; the Adriatic-facing part of the Balkan Peninsula, from Slovenia to Greece; the Massif Central Region in France; and a broad area encompassing Galicia, Castile and León (Spain) and northern Portugal (Fig. 5). It is, however, noteworthy that *C. piperata* has successfully established precisely in those areas that already exhibited the most favourable environmental conditions for the species. A further natural expansion of its range in northern Italy and into adjacent suitable regions can therefore be expected, possibly following patterns similar to those of two other non-native species of eastern Palaearctic origin, *Colasposoma dauricum* (Mannerheim, 1849) (Chrysomelidae) and *Psacothea hilaris* (Pascoe, 1857) (Cerambycidae), which have also become successfully established in northern Italy (Lupi et al. 2023, Magoga and Montagna 2023). The discovery of *C. piperata* in Italy, particularly the presence of an established population, is clearly linked to the country's central role in global trade, serving as a crossroads for import and export routes to and from Europe. This strategic position, combined with the remarkable diversity of climates, habitats and environments, including highly anthropogenised ones, that characterise the Italian territory, may favour the establishment and subsequent naturalisation of non-native species. In the absence of genetic data, however, it is not possible to determine whether the introduction of *C. piperata* resulted from a direct arrival from its native Asian range or from an indirect introduction mediated by populations already established in the Americas or in other regions of establishment where it may still be undetected. Currently, both scenarios are considered equally plausible. It should also be noted that Italy is often amongst the first European countries where new non-native species are reported. This does not necessarily imply that the country represents the actual area of first arrival, but rather reflects the presence of an active and widespread entomological community, coupled with a growing participation in citizen-science initiatives. This context substantially increases the likelihood of early detection of non-native species, improving the timeliness and quality of data available for research and for the management of biological invasions.

## Supplementary Material

XML Treatment for Cassida piperata

19DB5CDB-21F7-5ED6-949F-4D299504660C 10.3897/BDJ.13.e177770.suppl1 Supplementary material 1 Cassida piperata in Italy from iNaturalist Data type faunistic records Brief description Records of *Cassida piperata* in Italy available from iNaturalist (1 November 2025). File: oo_1461072.csv https://binary.pensoft.net/file/1461072 downloaded from iNaturalist

20AA2688-0E8D-57B5-A287-7EE310416538 10.3897/BDJ.13.e177770.suppl2 Supplementary material 2 Supplementary table Data type SDM outputs Brief description This file contains the performance of each model in the SDM pipeline (S1) and the variable importance (S2). File: oo_1493338.xlsx https://binary.pensoft.net/file/1493338 E. Ruzzier
